# Innate immune role of IL-6 in influenza a virus pathogenesis

**DOI:** 10.3389/fcimb.2025.1605446

**Published:** 2025-07-07

**Authors:** Xinxin Li, Chen Huang, Kul Raj Rai, Quanming Xu

**Affiliations:** ^1^ Meizhouwan Vocational Technology College, Putian, China; ^2^ Hanjiang District Agriculture and Rural Affairs Bureau, Putian, China; ^3^ Key Laboratory of Fujian-Taiwan Animal Pathogen Biology, College of Animal Sciences, Fujian Agriculture and Forestry University, Fuzhou, China; ^4^ Scientific Research and Experiment Center, Fujian Police College, Fuzhou, China

**Keywords:** IL-6, IAV, innate immunity, inflammation, cytokine storm

## Abstract

Interleukin-6 (IL-6), a pleiotropic cytokine, is induced by infection of influenza A virus (IAV), where it plays a pivotal role in immune defense and the regulation of inflammation. IAV induces IL-6 transcription upon recognition by pattern recognition receptors, which activate downstream signaling cascades, leading to the activation of transcription factors. Activated transcription factors subsequently regulate the expression of IL-6 in innate immune cells, such as macrophages, dendritic cells, and epithelial cells. IL-6 contributes to antiviral immunity by promoting the recruitment of immune cells to sites of infection and amplifying the inflammatory response. While optimal IL-6 production is essential for effective anti-IAV immunity, excessive IL-6 production can contribute to a dysregulated immune response, leading to a cytokine storm. In this context, IL-6 signaling, in coordination with other proinflammatory cytokines such as TNF-α and IL-1β, not only enhances its own production but can also serve as a key mediator of inflammation. This cascade can result in exaggerated immune responses, causing tissue damage and potentially leading to severe outcomes, including organ failure and death. Understanding the molecular mechanisms underlying cytokine storms presents important therapeutic opportunities. However, the precise pathways responsible for excessive IL-6 production and its dysregulation during IAV infection is not fully understood. This review explores the reported mechanisms regulating IL-6 induction in response to IAV and its innate immune role in IAV pathogenesis, highlighting existing research gaps in understanding IAV-induced IL-6 production and its impact on immune modulation. A deeper understanding of IAV-induced IL-6 production and signaling could inform the development of targeted therapies to more effectively manage influenza.

## 1 Introduction

Influenza A virus (IAV) is a highly contagious respiratory pathogen responsible for seasonal flu outbreaks and periodic pandemics, posing a significant global health burden (Monto and Fukuda, 2020). A member of orthomyxoviridae family, IAV is characterized by its segmented RNA genome, which enables frequent genetic reassortment and rapid mutation, allowing it to evade immune surveillance (Shao et al., 2017). IAV encodes multiple structural and nonstructural proteins, such as hemagglutinin (HA), neuraminidase (NA), nucleoprotein (NP), components of the RNA polymerase complex including polymerase basic 1 (PB1) and PB2, matrix 1 (M1), and nonstructural protein 1 (NS1) (Krammer et al., 2018). The virus infects epithelial cells of the respiratory tract, causing symptoms ranging from mild illness to severe complications such as viral pneumonia, acute respiratory distress syndrome (ARDS), and multi-organ failure (Kalil and Thomas, 2019). The World Health Organization estimates that IAV infects nearly 10% of the world’s population annually, resulting in approximately 3 to 5 million cases of severe illness and between 290,000 to 650,000 respiratory-related deaths worldwide (Uyeki et al., 2022). The most devastating “Spanish flu” pandemic in 1918 took over 50 million lives. The subsequent emergence of flu pandemics, such as “Asian flu” and “Hong Kong flu” in 1957 and 1968, respectively, killed about three million people (Krammer et al., 2018; Rai et al., 2021). The continuous antigenic evolution of IAV presents an ongoing challenge for vaccine development and antiviral strategies, underscoring the need for a deeper understanding of host immune responses to the virus for alternative therapeutics.

The immune response to IAV consists of both innate and adaptive immunity, with the innate immunity playing a crucial role in the initial defense against viral invasion (Rai et al., 2021). Upon infection, pattern recognition receptors (PRRs) such as Toll-like receptors (TLRs) and retinoic acid-inducible gene I (RIG-I)-like receptors (RLRs) detect viral components, leading to the activation of signaling pathways that stimulate the production of interferons (IFNs) and pro-inflammatory cytokines, including Interleukin-6 (IL-6) (Carty et al., 2021). This early immune response is vital for controlling viral replication and promoting the activation of the adaptive immune system, which provides long-term immunity (Kawai and Akira, 2010). However, an excessive or dysregulated immune response can contribute to immunopathology, exacerbating tissue damage and inflammation. Understanding the balance between protective and detrimental immune responses is critical for developing effective therapeutic strategies against IAV infections.

During IAV infection, cytokines mediate antiviral defenses and immune cell recruitment (Chan and Gack, 2016; Chen et al., 2017). PRRs detect pathogen-associated molecular patterns (PAMPs), such as viral RNA and proteins, as well as damage-associated molecular patterns (DAMPs) from infected host cells (Pillai, 2014; Chen et al., 2018; Ramos et al., 2019). Key PRRs—including TLR, RLR, and NLR (NOD-like receptor) pathways—enable influenza recognition, triggering antiviral and inflammatory responses (Tanaka et al., 2014). TLRs, such as TLR3, TLR7, and TLR8, are membrane-bound PRRs that recognize viral RNA: TLR3 detects double-stranded RNA (dsRNA), a byproduct of influenza replication, TLR7/8 sense single-stranded RNA (ssRNA) from the viral genome, while TLR2 and TLR4 recognize influenza-induced DAMPs (Shinya et al., 2012; Kim et al., 2022). TLRs activate signaling cascades *via* immune adaptor proteins like MyD88 or TRIF, leading to the production of type I IFN-α/β and pro-inflammatory cytokines (e.g., IL-6, TNF-α) through transcription factors like nuclear factor kappa-light-chain-enhancer of activated B cells (NF-κB) and interferon regulatory factors (IRFs) (Iwasaki and Pillai, 2014; Maarouf and Rai, 2018). This response is critical for early antiviral defense and immune cell recruitment (Pillai, 2014).

In the cytoplasm, RLRs, including RIG-I and MDA5, act as PRRs to detect viral RNA (Pillai, 2014). RIG-I recognizes short dsRNA and 5’-triphosphate RNA, while MDA5 senses long dsRNA (Chan and Gack, 2015). In influenza virus induced RLRs signaling, the mitochondrial antiviral-signaling protein (MAVS) plays a key role as immune adaptor protein that not only activates the downstream protein kinase TANK-binding kinase 1 (TBK1), but also license TBK1 to phosphorylate transcriptional factor IRF3, triggering the production of interferons (Gaur et al., 2011; Jensen and Thomsen, 2012; Pillai, 2014; Liu et al., 2015; Hagenaars et al., 2016). The NLR pathway, particularly NLRP3, is another cytosolic PRR that detects cellular stress or damage caused by influenza infection, often in response to PAMPs like viral proteins or DAMPs such as ATP or reactive oxygen species (ROS) (Niu and Meng, 2023). NLRP3 forms an inflammasome complex, activating caspase-1, which processes pro-IL-1β and pro-IL-18 into their active forms, driving inflammation and immune cell recruitment (Negash et al., 2013; Guo et al., 2015; Niu and Meng, 2023).

Among the various cytokines involved in the innate immune response to IAV, IL-6 stands out as a pivotal player in the innate immune response to IAV infection (Gentile et al., 1998; Thomas et al., 2009; Chiaretti et al., 2013; Richetta and Faure, 2013; Killip et al., 2015; Yang et al., 2017; Kalil and Thomas, 2019; Choy et al., 2020; Carty et al., 2021). IL-6, a pleiotropic cytokine with diverse functions, was identified in 1973 as a soluble factor secreted by T cells (Choy et al., 2020). Over the past 50 years, it has become recognized as a crucial immune regulator, modulating acute phase responses, promoting B and T cell differentiation, and regulating inflammatory processes (Grebenciucova and VanHaerents, 2023). It is primarily produced by macrophages, dendritic cells, and epithelial cells in response to viral infections and other inflammatory stimuli (Grebenciucova and VanHaerents, 2023). IL-6 exerts its effects through binding to the IL-6 receptor (IL-6R) and subsequent activation of the Janus kinase/signal transducer and activator of transcription (JAK/STAT) pathway, leading to the expression of genes involved in immune regulation (Grebenciucova and VanHaerents, 2023).

Despite its the role in antiviral defense, IL-6 exhibits a dual nature in the immune response to IAV, with both protective and detrimental effects (Gentile et al., 1998; Bradley-Stewart et al., 2013; Chiaretti et al., 2013, Chi et al, 2013; Radigan et al., 2019; Zuo et al., 2021; Zhang et al., 2022; Grebenciucova and VanHaerents, 2023). On one hand, IL-6 contributes to viral clearance by promoting the activation and proliferation of immune cells, enhancing antibody production, and supporting tissue repair (Tanaka et al., 2014; Zuo et al., 2021). Studies have shown that IL-6-deficient mice exhibit impaired immune responses to IAV, suggesting a protective role in host defense (Dienz et al., 2012; Lauder et al., 2013; Yang et al., 2017). On the other hand, excessive IL-6 production has been associated with severe inflammatory responses and immunopathology, particularly in cases of hypercytokinemia or ‘cytokine storm’ (Fajgenbaum and June, 2020; Gu et al., 2021). In severe influenza infections, elevated IL-6 levels correlate with increased disease severity, lung inflammation, and poor clinical outcomes (Paquette et al., 2012; Radigan et al., 2019; Gu et al., 2021). Thus, dysregulated IL-6 can contribute to respiratory failure and an increased risk of mortality.

Given the complex and context-dependent role of IL-6 in IAV infection, a comprehensive understanding of its functions is essential for developing targeted therapeutic approaches. This review discusses the mechanisms of IAV-induced IL-6 production and IL-6 signaling pathways, emphasizing its potential as an inflammation amplifier that may contribute to a dysregulated immune response during IAV infection. We also examine the dual role of IL-6 in IAV pathogenesis. A deeper understanding of IL-6 regulation within the innate immune response could offer critical insights for improving treatment strategies for influenza infections.

## 2 Cytokine storm in influenza

Cytokines play a dual role in influenza infection (Jiang and Zhang, 2023). Initially, they are crucial for viral clearance by promoting immune cell recruitment and activation (Zuo et al., 2021). IL-6, TNF-α, and IL-1β enhance the infiltration of neutrophils and monocytes into infected tissues, aiding in viral elimination (Dienz et al., 2012; Lauder et al., 2013). Chemokines such as CXCL10 and CCL2 further mediate immune cell trafficking to the lungs (Alon et al., 2021). However, excessive cytokine production can be detrimental, leading to immune dysregulation and severe tissue damage. A cytokine storm refers to the excessive and uncontrolled release of pro-inflammatory cytokines, which can cause significant immunopathology (Fajgenbaum and June, 2020; Gu et al., 2021; Jiang and Zhang, 2023). In severe influenza cases, including those caused by highly virulent strains such as H5N1 and H1N1, an overwhelming cytokine response leads to widespread inflammation, lung injury, and ARDS (Short et al., 2017; Kalil and Thomas, 2019). The cytokine storm results in increased vascular permeability, leading to pulmonary edema and impaired gas exchange (Chen et al., 2018; Jiang and Zhang, 2023). Overactivation of immune cells, causing collateral damage to lung tissue. Dysfunctional coregulation pathways, increasing the risk of thrombosis and multi-organ failure (Vincent et al, 2019). The severity of influenza is often correlated with the magnitude of the cytokine storm, and individuals with heightened immune responses may suffer from more severe disease outcomes (Bradley-Stewart et al., 2013; Vincent and Tang BM, Cootes T, 2019; Fajgenbaum and June, 2020). Excessively elevated levels of cytokines such as IFN-γ, IL-6, IL-1α, IL-1β, TNF-α, IL-15, IL-12p70, IL-17, IL-10, MCP-1, MIP-1β, IL-8, MIG, IP-10, MIP-1α, GM-CSF, and RANTES were observed in cytokine storm caused by H1N1 (“Pandemic strain”) influenza virus (reviewed in (Morris et al., 2021)). Among the inflammatory cytokines, IL-6 is a critical cytokine contributing to cytokine storm in the pathogenesis of IAV virus. For a more detailed understanding of the mechanisms underlying the cytokine storm induced by influenza virus, readers can refer to review articles (Gu et al., 2019, Gu et al, 2021).

## 3 Roles of IL-6 in innate immune response to IAV

IL-6 plays a crucial role in innate immunity, particularly in response to IAV. It contributes to antiviral defense, inflammation regulation, and immune cell activation. While essential for host defense, excessive IL-6 production can lead to pathological inflammation, highlighting its dual and complex role in immunity.

### 3.1 IL-6 Induction in response to IAV

The upregulation of IL-6 during IAV infection has been reported since the late 1980s, with further characterization in the 1990s (Sprenger et al., 1994; Pahl and Baeuerle, 1995; Matsukura et al., 1996; Gentile et al., 1998; Visseren et al., 1999). IL-6 is induced in response to various subtypes of IAV infection by both immune and non-immune cells, including macrophages, dendritic cells, endothelial cells, and epithelial cells (Matsukura et al., 1996; Hn et al., 2013; Short et al., 2017; Yang et al., 2017; Gou et al., 2020; Gu et al., 2021; Xie et al., 2021). The production of IL-6 is primarily triggered by the activation of PRRs, such as TLRs and RLRs, which recognize viral components and DAMPs (Shinya et al., 2012; Prantner et al., 2017; Chen et al., 2018; Liu et al., 2019; Gu et al., 2021; Jiang and Zhang, 2023). Among TLRs, the role of TLR9 in IAV-induced DAMP recognition remains less clear, studied only in the co-infection model (Tuvim et al., 2012; Maarouf et al., 2018; Sun and Metzger, 2019). RLRs (RIG-I and MDA5), on the other hand, sense viral RNA within the cytoplasm and initiate antiviral responses. Upon activation, these PRRs trigger intracellular signaling cascades, including the NF-κB, AP-1, and MAPK (ERK, JNK, p38) pathways (Eastman et al., 1996; Brydon et al., 2005; Klemm et al., 2017). NF-κB and AP-1 are the primary transcription factors driving IAV-induced IL-6 gene expression, while MAPK signaling further enhances cytokine production by promoting transcription factor phosphorylation (Visseren et al., 1999; Ludwig et al., 2001; Pinto et al., 2011; Liu et al., 2019; Gu et al., 2021).

The magnitude of cytokine induction, including IL-6, in IAV infection is determined by a multifactorial interplay involving influenza strain specificity, viral protein polymorphisms, replication kinetics, and host-specific immune responses (Li et al., 2006, Li et al, 2018; Newby et al., 2007). Cumulative evidence suggests that certain influenza virus strains induce higher levels cytokines including IL-6 and are more prone to triggering cytokine storms, with pathogenicity linked to strain-specific viral protein functions (De Jong et al., 2006; Perrone et al., 2008; Hn et al., 2013; Li et al., 2018). For example, highly pathogenic strains such as H5N1 and the 1918 H1N1 pandemic virus elicit elevated IL-6 production and severe immunopathology compared to seasonal H1N1 strains (De Jong et al., 2006; Boon et al., 2011; Kang et al., 2011; Kalaiyarasu et al., 2016; Li et al., 2018). Among IAV proteins, the NS1 protein is a potent inhibitor of cytokine production and innate immune signaling (García-Sastre et al., 1998; Marc, 2014; Rai et al., 2020), which exhibits strain-specific sequence differences (Hale et al., 2008). NS1 mutation affects IAV replication efficiency by modulating cytokine expression (Newby et al., 2007; Chen et al., 2021a). Of note, strains with higher replication efficiency generate increased viral loads (Boon et al., 2011; Li et al., 2018). Elevated viral loads can hyperactivate PRRs, boosting IL-6 overproduction and increasing the risk of cytokine storm (Li et al., 2018). Certain strain-specific IAV proteins, including HA (Pahl and Baeuerle, 1995), NA (Kim et al., 2022), M1 (Kim et al., 2023), PB1-F2 (Zeng et al., 2021), contribute to IL-6 production by activating the NF-κB signaling pathway, while dampening IFN-mediated antiviral responses (Kochs et al., 2007; Iwai et al., 2010; Zeng et al., 2021; Zhang et al., 2023; Ouyang et al., 2024), indicating polymorphisms, or variations in these viral proteins, may lead to differential cytokine responses (Newby et al., 2007; Williams et al., 2024). Moreover, other factors such as host factors and receptor tropism also influence immune activation resulting into differential IL-6 induction in the response to IAV (reviewed in (Long et al., 2019)).

**Table 1** summarizes representative evidence regarding IL-6 induction by IAV and viral components, such as HA and NP, across various experimental models, including human cell cultures, animal models, and human populations. The data demonstrate that IL-6 is significantly upregulated during infections with different IAV strains, including H5N1 and H7N9, contributing to proinflammatory responses, viral clearance, and tissue protection. Elevated IL-6 levels are often correlated with the severity of infection. Furthermore, IAV-induced IL-6 exerts an immunomodulatory role in both respiratory and extra-respiratory tissues, including the central nervous system.

**Table 1:** Early and some recent representative evidences for IL-6 upregulation following IAV infection.

SN	Virus strain/viral components	Model	Description	References
1.	IAV	Human monocytes	Significant upregulation of IL-6 following IAV infection.	(Sprenger et al., 1994)
2.	IAV HA	Hela and 293T Cells	Viral HA activates transcription factor NF-kB.	(Pahl and Baeuerle, 1995)
3.	IAV (H3N2)	Bronchial epithelial cells	Significant upregulation of IL-6 following IAV infection.	(Matsukura et al., 1996)
4.	IAVA/Kawasaki/86 (H1N1)	Healthy human population	Increased IL-6 levels in nasal lavage samples following experimental IAV infection.	(Gentile et al., 1998)
5.	1997–1998 flu season IAV strains	Human population	Elevated levels of IL-6 in cerebrospinal fluid and serum samples associated with influenza virus-induced neurological disorders in children	(Ito et al., 1999; Aiba et al., 2001)
6.	H1N1	Human population	IL-6 plasma levels were significantly elevated in patients with H1N1 infection, with higher levels in severe cases. No correlation was found between IL-6 levels and clinical outcomes.	(Chiaretti et al., 2013)
7.	pH1N1 and fatal HPAI H5N1 virus	Ferrets	Upregulation of IL-6 in extra-respiratory tissues, particularly the CNS, contributes to proinflammatory cytokine responses during severe pH1N1 and fatal H5N1 infections.	(Short et al., 2017)
8.	1918 H1N1 influenza virus,	Ferrets	Proinflammatory cytokines, including IL-6, were upregulated in respiratory and extra-respiratory tissues (e.g., olfactory bulb, spinal cord, liver, heart, pancreas)	(De Wit et al., 2018)
9.	H1N1 viruses, H5N1/97 and H5N1/04 viruses	Primary human alveolar and bronchial epithelial cells	H5N1/97 and H5N1/04 viruses were more potent inducers of IL-6 than H1N1 viruses *in vitro*.	(Chan et al., 2005)
10.	IAV (H1N1) pdm09	Human population	Cytokine responses in patients with mild or severe influenza A (H1N1) pdm09	(Bradley-Stewart et al., 2013)
11.	Avian influenza H5N1 virus	Chicken monocyte-derived dendritic cells	Cytokines including IL-6 were significantly upregulated in the H5N1 cells.	(Kalaiyarasu et al., 2016)
12.	H7N9	Human population	IL-6 was one of the most upregulated cytokines in virus infected patients.	(Chi et al., 2013)
13.	A/Puerto Rico/8/1934 (H1N1), H5N3, H5N1, H7N9	Airway epithelial and immune cells (mammalian and avian)	H5N1 and H7N9 viruses significantly stimulated IL-6	(Ye et al., 2015)
14.	IAV/Hong Kong/498/97 (H3N2)	Human cells	IAV infection induces soluble interleukin-6 receptor upregulation and that mediates the IL-6 and IL-32 inflammatory cytokine burst.	(Wang et al., 2015)
15.	H1N1 Influenza Virus	Human population	IL-6 upregulation in children with influenza virus infection	(Chiaretti et al., 2013)
16.	H1N1 strains including A/WSN/33 (WSN), A/PR/8/34 wild type (PR8) and A/CA/04/09 (CA04)	Mice and Cell model	IAV-induced robust expression of SOCS3 contributes to excessive production of IL-6	(Liu et al., 2019)
17.	Influenza A/Puerto Rico/8/1934 (PR8)	Mice Model	Extracellular viral NP interacts with TLR2 and TLR4 to induce proinflammatory cytokine including IL-6	(Kim et al., 2022)
18.	IAV M1	Cells and Mice Model	IAV M1 protein treatment led to robust expression of IL6	(Kim et al., 2023)

### 3.2 IL-6 signaling pathways: classical and trans-signaling

**Figure 1:**
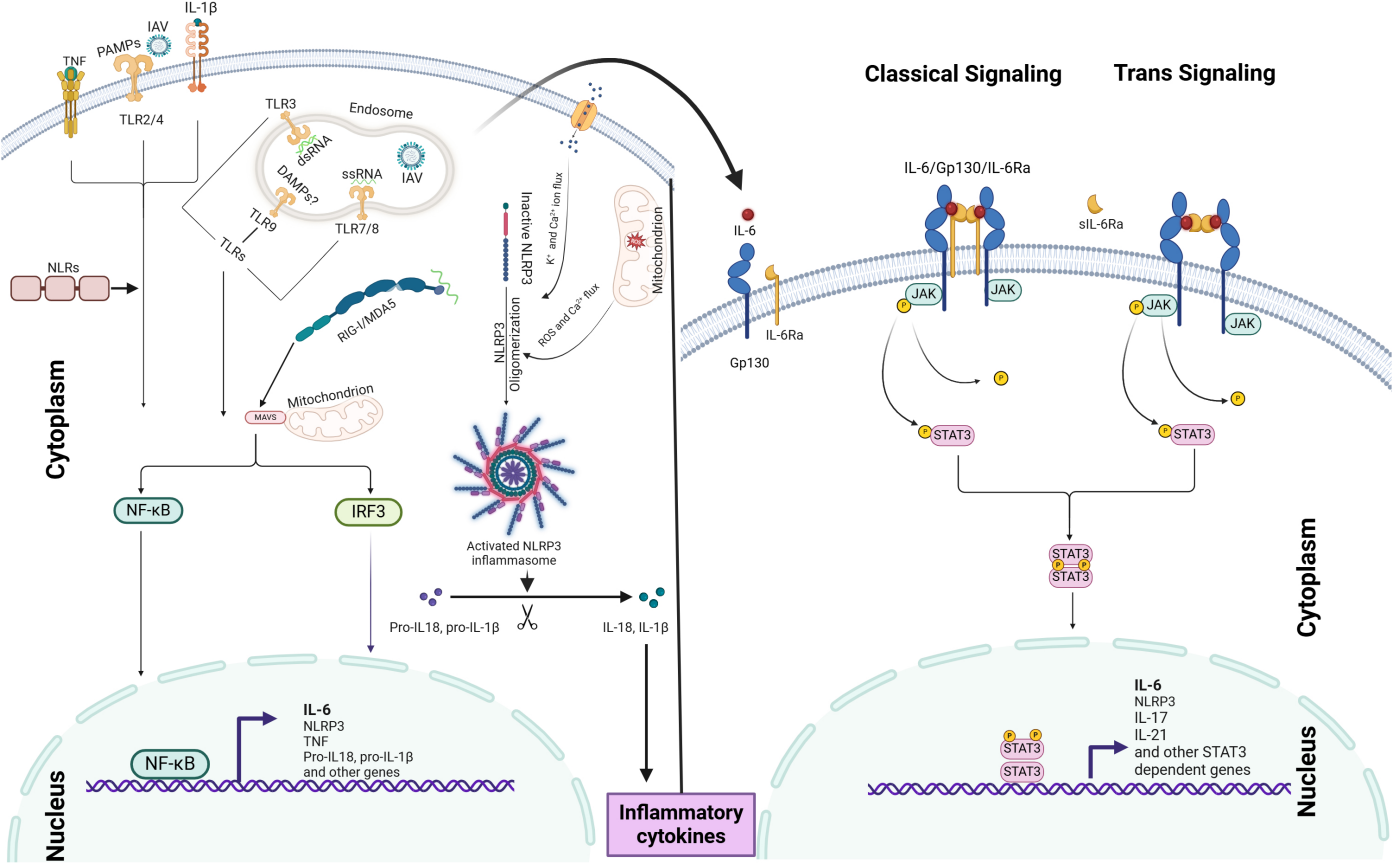
IAV-induced IL-6 induction mechanism and IL-6 signaling pathways: IL-6 production is initiated by PRRs, such as TLRs and RLRs, which recognize viral PAMPs and DAMPs, leading to the activation of NF-κB signaling and IL-6 expression. The NLRP3 inflammasome further amplifies IL-6 production through IL-1β and IL-18 release. IAV-induced IL-6 modulates immune responses *via* two distinct signaling pathways: (i) classical signaling, involving membrane-bound IL-6R, and (ii) trans-signaling, mediated by soluble IL-6R. Ligand binding to these receptors activates the JAK-STAT3 pathway, driving downstream gene expression. The coordinated activation of both NF-κB and JAK-STAT3 pathways acts as an inflammation amplifier.

IAV-induced IL-6 orchestrates innate immune responses through two distinct IL-6 signaling pathways: (i) classical signaling, which involves membrane-bound IL-6 receptor (mIL-6R), and (ii) trans-signaling, which relies on the soluble IL-6 receptor (sIL-6R). Binding of the ligand to the receptor activates a cascade of events that regulate downstream gene expression and trigger various cellular responses, including inflammation (Rose-John, 2012; Tanaka et al., 2014; Wang et al., 2015; Pullamsetti et al., 2018; Radigan et al., 2019; Velazquez-Salinas et al., 2019) (Please also refer the **Figure 1** below).

IL-6 classical pathway: The activation of the IL-6 receptor begins when IL-6 binds to IL-6Rα, which causes a conformational change in the receptor complex. This change allows for the recruitment and dimerization of gp130, forming a hexameric signaling complex (Pullamsetti et al., 2018). The hexamerization of gp130 triggers intracellular signaling by inducing phosphorylation events and activating downstream signaling pathways, such as JAK/STAT, MAPK/ERK, and PI3K/AKT (Rose-John, 2012; Pullamsetti et al., 2018). These pathways play a crucial role in mediating IL-6-induced gene expression, cellular proliferation, differentiation, survival, and immune responses.

IL-6 Trans signaling pathway: This pathway involves the soluble form of the IL-6 receptor (sIL-6R) and gp130 (Wang et al., 2015). It enables IL-6 to influence cells that do not express the membrane-bound IL-6 receptor (mIL-6R). The trans signaling pathway is initiated when IL-6 binds to sIL-6R in the extracellular space. The IL-6/sIL-6R complex can then diffuse and reach cells lacking the mIL-6R. When it encounters such cells, the complex binds to the ubiquitously expressed gp130 on their surfaces (Rose-John, 2012; Liu et al., 2024). This binding induces conformational changes in gp130 that activate JAKs associated with the receptor’s cytoplasmic domain. Activated JAKs phosphorylate specific tyrosine residues on gp130, creating docking sites for downstream signaling molecules.

In the STAT pathway, phosphorylated gp130 serves as a docking site for STAT proteins, particularly STAT3 (Zhang et al., 2000). Upon recruitment to the phosphorylated gp130, JAK1 and JAK2 carry out phosphorylation. Once phosphorylated, STAT3 forms either homodimers or heterodimers with other STAT isoforms and translocates from the cytoplasm to the nucleus (Liu et al., 2024). Within the nucleus, STAT3 dimers bind to specific DNA sequences called STAT response elements located in the promoter regions of target genes (Arshad et al., 2020), driving expression of various STAT3 dependent genes.

### 3.3 IL-6 amplifier and IL-6-mediated crosstalk in IAV infection

An IL-6 amplifier, also referred to as an inflammation amplifier, comprises molecules, receptors, or signaling pathways that enhance IL-6 activity (Lee et al., 2012). This system establishes a self-perpetuating inflammatory cycle through the coordinated activation of IL-6, NF-κB, and STAT3 signaling (Lee et al., 2012). As illustrated in the **Figure 1**, upon IAV infection, viral components are detected by PRRs, initiating the first wave of IL-6 production *via* NF-κB activation. The secreted IL-6 then binds to its receptor (IL-6R), activating JAK-STAT3 signaling, which promotes the expression of additional inflammatory mediators, including TNF and IL-1β. These secondary cytokines reinforce feed-forward loops that further stimulate IL-6 production through autocrine and paracrine mechanisms, potentially resulting in excessive cytokine release and tissue injury (Gu et al., 2021; Niu and Meng, 2023).

The IL-6-mediated crosstalk during IAV infection is intricate (Thomas et al., 2009; Malik and Zhou, 2020). Ligand-receptor complex formation *via* either classic or trans-signaling pathways trigger the activation of multiple intracellular signaling cascades, including the JAK-STAT pathway, Ras-MAPK pathway, p38 and JNK MAPK pathways, PI3K-Akt pathway, and the MEK-ERK5 pathway (Klemm et al., 2017). Notably, both classic and trans-signaling complexes activate similar signaling pathways (Liu et al., 2024). NF-κB and STAT3 cooperatively regulate the expression of several inflammatory cytokines, including IL-6 itself, as well as other cytokines like TNF, CCL2, CCL5, IL-8, and IL-17 and so forth. Additionally, they modulate the expression of inflammation amplifiers such as NLRP3, further intensifying the inflammatory response (Pandey et al., 2023).

Although, NLRs (NOD1 and NOD2) are not primary sensors of IAV but can upregulate inflammation *via* crosstalk with other PRRs and inflammatory signaling pathways like NF-κB and the NLRP3 inflammasome. Importantly, the NLRP3 inflammasome plays a critical role in IL-6 production and amplification during IAV infection. Its activation involves two sequential steps: the priming signal, triggered by viral recognition *via* PRRs, leading to the upregulation of NLRP3, as well as the transcription of pro-IL-1β and pro-IL-18. Subsequently, viral components or host-derived DAMPs induce the activation of NLRP3, resulting in the oligomerization of the inflammasome mechanisms such as the ion channel activity of the IAV M2 protein, mitochondrial ROS production, and disruption of cellular homeostasis (Zamarin et al., 2006; Thomas et al., 2009; Ichinohe et al., 2010; Pinar et al., 2017; Niu and Meng, 2023) (please refer the **Figure 1** below). This leads to caspase-1 activation and subsequent IL-1β and IL-18 release, which can further amplify IL-6 production by binding with their cell membrane bound receptors resulting into the activation of NF-κB (Chanturiya et al., 2004; Thomas et al., 2009; Ichinohe et al., 2010; Kinoshita et al., 2015; Zeng et al., 2021; Kim et al., 2022) (see **Figure 1**).

In parallel, the PI3K-Akt pathway can also contribute to IL-6 induction, with viral proteins such as hemagglutinin and non-structural protein 1 triggering Akt phosphorylation (Ehrhardt et al., 2007), which sustains NF-κB activation and IL-6 transcription (Arora et al., 2016). Although IRF3 and IRF7 primarily drive type I interferon responses (Chen et al., 2018), IFNs can also secondarily amplify IL-6 production through autocrine and paracrine signaling mechanisms (Murray et al., 2015). The relative contribution of these pathways varies across different cell types; for instance, bronchial epithelial cells predominantly rely on TLR3 and TLR7, whereas macrophages primarily utilize RLRs. For example, bronchial epithelial cells predominantly use TLR3 and TLR7, while macrophages rely more on RLRs (Malik and Zhou, 2020).

### 3.4 The dual role of IL-6 in influenza infection

IL-6 has a dual role in influenza infections, exerting both protective and detrimental effects (Longhi et al., 2008; Vogel et al., 2014; Hn et al., 2013; Zuo et al., 2021). It facilitates viral clearance by enhancing the innate immune response, aids in lung tissue repair, and regulates immune cell (T- and B-cell) activity to modulate inflammation and promote protective immunity (Dienz et al., 2012; Velazquez-Salinas et al., 2019). Additionally, IL-6 supports neutrophil function and preserves epithelial integrity, thereby limiting viral replication and preventing secondary bacterial infections (Dienz et al., 2012). However, excessive IL-6 production can lead to immune dysregulation and a cytokine storm, which exacerbates disease severity (Yu et al., 2011; Bradley-Stewart et al., 2013; Short et al., 2017). Elevated IL-6 levels are associated with poor outcomes, including ARDS and increased mortality (Bradley-Stewart et al., 2013; Short et al., 2017). In co-infection scenarios, dysregulated IL-6 exacerbates lung damage and impairs bacterial containment (Kalil and Thomas, 2019; Gou et al., 2020). However, its effects are context-dependent. **Table 2** presents a brief summary when IL-6 is beneficial versus when it becomes detrimental.

**Table 2:** Beneficial vs. detrimental effects of IL-6 in influenza infection (please refer **Supplementary Table 1** for more detail).

SN	Context	Beneficial effects	Detrimental Effects
1.	Immune Response	➢ Mounts early antiviral innate immune response (Lauder et al., 2013) (Zuo et al., 2021). ➢ Enhances antiviral cellular responses (Leethese et al., 1999; Dienz et al., 2012) ➢ Promotes protective antibody production and viral clearance (Piattini et al., 2025).	➢ Mediates excessive inflammation (inflammation amplifier) (Gu et al., 2019). ➢ Contributes to cytokine storm (Gu et al., 2021). ➢ Leads to viral persistence and tissue damage (Wang et al., 2010).
2.	Tissue Protection	Preserves lung epithelial integrity (Yang et al., 2017)	Promotes muscle wasting (Radigan et al., 2019)
3.	Long-term Immunity	Boosts memory CD4+ T-cell formation (Leethese et al., 1999; Strutt et al., 2016)	Not specified
4.	Co-infections	Enhances macrophage bacterial clearance (Gou et al., 2020)	Worsens secondary infections (Klemm et al., 2017)

#### 3.4.1 Host protective function of Il-6

Multiple lines of evidence support that IL-6 protects the host against influenza by enhancing antiviral responses. Studies using IL-6 knockout models suggest that IL-6 boosts both innate and adaptive immune responses to influenza. For instance, IL-6 was shown to be crucial for resolving infection by protecting neutrophils from virus-induced death and enhancing their viral clearance activity (Dienz et al., 2012). IL-6 knockout resulted in prolonged viral persistence in the lung, leading to severe damage and eventual death (Dienz et al., 2012). Mice lacking IL-6 exhibited a profound defect in mounting an anti-viral T-cell response, accompanied by increased infiltration of inflammatory monocytes and elevated production of pro-inflammatory cytokines like IFN-α and TNF-α (Lauder et al., 2013). Early IL-6 signaling was also shown to promote IL-27-dependent maturation of regulatory T cells, aiding in the resolution of viral immunopathology (Pyle et al., 2017). Additionally, IL-6 has been suggested to facilitate lung repair during influenza infection (Yang et al., 2017).

A study also demonstrated that IL-6 interacts with IL-27 to form IL-6–IL-27 complexes, which exhibit antiviral activity. These complexes enhance the expression of influenza-induced inflammatory cytokines, IFNs, and ISGs, while promoting the phosphorylation of STAT1 and STAT3 (Zuo et al., 2021). In an IAV-*Streptococcus pneumoniae* co-infection model, IL-6 was shown to reduce cell death in the lungs and enhance bacterial phagocytosis through upregulation of MARCO expression in macrophages, limiting infection-related inflammation and controlling bacterial invasion (Gou et al., 2020). Overall, it appears that early and optimal IL-6 production is beneficial for host protection. However, IL-6 must also be tightly regulated during the early immune response to balance effective immunity and inflammation. Additional host-protective functions of IL-6 in influenza virus infection are summarized in **Supplementary Table 1**.

#### 3.4.2 Detrimental role of IL-6 in influenza virus infection

In certain cases, especially during severe influenza infections, IL-6 can contribute to a hyper-inflammatory state known as a “cytokine storm.” This is characterized by an excessive release of pro-inflammatory cytokines that can cause tissue damage, organ failure, and even death. In severe influenza infections, IL-6, along with other cytokines like TNF-α and IL-1β, can exacerbate the disease, leading to complications such as ARDS. For example, fatal cases of human influenza A H5N1 exhibited elevated IL-6 levels, indicating IL-6-mediated hypercytokinemia, which contributed to reactive hemophagocytic syndrome, multiorgan failure, and ultimately, death (To et al., 2001). IL-6 promoted muscle degradation via JAK/STAT, FOXO3a, and atrogin-1 upregulation and the treatment with treatment with a Food and Drug Administration-approved Ab antagonist to the IL-6R (tocilizumab) attenuated the severity of IAV-induced muscle dysfunction (Radigan et al., 2019). Another study has shown that the influenza virus-cytokine-protease cycle as one of the main mechanisms for vascular dysfunction in severe influenza (Wang et al., 2010). IL-6 can act as a bridge between innate immunity and oxidative stress, leading to worsened pathology. For example, inactivated influenza virus induces the production of oxidized phospholipids through the TLR4-IL-6-ROS signaling cascade, contributing to acute lung injury (Imai et al., 2008). Moreover, immunocompromised individuals, particularly the elderly, are prone to elevated risk of severe influenza, largely driven by IL-6-mediated hyperinflammation (Harpur et al., 2021). Notably, an IL-6 amplifier mechanism appears crucial in the pathogenic cascade triggered by dysregulated IL-6 signaling in IAV infection. Other representative pathogenic implications IAV-induced IL-6 is described in the **Supplementary Table 1**.

## 4 Clinical and therapeutic implications

### 4.1 IL-6 as a biomarker

IL-6 has been shown as a potential biomarker for assessing influenza severity, with elevated levels correlating with disease progression and poor clinical outcomes (Ito et al., 1999; Paquette et al., 2012). Studies have shown that patients with severe influenza, particularly those developing ARDS, exhibit significantly higher IL-6 levels compared to those with mild infections (Gentile et al., 1998; Kalil and Thomas, 2019). This positions IL-6 as a valuable prognostic and predictive biomarker, aiding in risk stratification and early identification of high-risk patients Beyond prognosis, IL-6 holds significant utility in guiding therapeutic development, as its levels can inform the efficacy and safety of anti-inflammatory interventions (Denisova et al., 2014; Tanaka et al., 2014; Radigan et al., 2019; Grebenciucova and VanHaerents, 2023). However, the acceptable threshold of IL-6 production remains debated, particularly in preclinical studies where biomarker standardization is critical but not yet fully established. The strong association between IL-6 and disease severity has spurred interest in targeting IL-6 or it signaling pathways, highlighting its dual role as both a biomarker and a therapeutic target in influenza and other inflammatory conditions.

### 4.2 Targeting IL-6 in influenza treatment

Targeting IL-6 signaling pathway is an important alternative therapeutic strategy for mitigating hyperinflammation in severe influenza cases. Although various therapeutic approaches have been attempted, completely blocking IL-6 would impair the innate immune responses (Kishimoto, 2005; Kang and Kishimoto, 2021). Instead, protection by IL-6 inhibition using short term antibodies to block is good therapeutic pathway to reduce the detrimental effect of IL-6 (Kishimoto, 2005; Narazaki and Kishimoto, 2018). Accordingly, anti-inflammatory drugs that target IL-6 signaling, such as monoclonal antibodies and Janus kinase (JAK) inhibitors, have been explored in various viral infections. Oclacitinib, a JAK inhibitor, and arbidol hydrochloride, known for its anti-inflammatory properties, are among the drugs studied in this context (Liu et al., 2013; Yu et al., 2024). Monoclonal antibodies like tocilizumab, which blocks the IL-6 receptor, have been particularly explored as potential treatments for reducing inflammation in severe cases of influenza (Tanaka et al., 2014; Radigan et al., 2019; Grebenciucova and VanHaerents, 2023). While IL-6 blockade has shown promise in conditions like rheumatoid arthritis (Davies and Choy, 2014) and COVID-19 (The REMAP−CAP Investigators, 2021). However, their application in influenza treatment has not been widely established. The main challenge lies in striking a balance between reducing harmful inflammation while preserving the essential antiviral functions of IL-6. Suppressing IL-6 aggressively may impair immune responses, potentially prolonging viral persistence or increasing susceptibility to secondary infections. Experimental trials evaluating IL-6 inhibitors in viral infections, including influenza and co-infections, have yielded mixed results (Pandey et al., 2023). While some studies indicate reduced inflammatory damage and improved respiratory function, others highlight concerns about increased risk of secondary bacterial infections due to immune suppression. Indeed, therapeutic targeting of IL-6 requires precise timing, as IL-6 levels rise early in infection, before peak viral clearance (Leethese et al., 1999; Chiaretti et al., 2013; Yang et al., 2017). Late inhibition (after viral peak) may improve outcomes by dampening excessive inflammation (Kishimoto, 2005; Choy et al., 2020; Kang and Kishimoto, 2021) but early blockade compromise antiviral immunity (Choy et al., 2020), suggesting most effective during the transition from viral replication to hyperinflammation. Future research should focus on refining therapeutic strategies, potentially using combination approaches that modulate IL-6 activity without entirely abolishing its protective effects (Pandey et al., 2023). Additionally, further clinical trials are needed to determine optimal patient selection, timing, and dosing for IL-6-targeted therapies in managing IAV infections.

## 5 Perspectives

IL-6, a key mediator of inflammation, is induced by the activation of innate immune responses during IAV infection. While optimal IL-6 production is beneficial for the host by promoting anti-IAV defenses, excessive IL-6 production can lead to hyperinflammation and contribute to a cytokine storm. Various amplifiers of IL-6, including NLRP3, play a critical role in amplifying inflammation through a positive feedback loop. However, the molecular mechanisms underlying the balance between optimal and excessive IL-6 production during IAV infection remain poorly understood. This complexity arises from the intricate and interconnected nature of innate immune signaling pathways, which involve crosstalk that is not yet fully elucidated.

During IAV infection, both PAMPs and DAMPs activate multiple signaling pathways. For instance, in co-infection models involving bacteria and IAV, enhanced activity of MAPKs such as p38 and ERK1/2 leads to excessive IL-6 production (Klemm et al., 2017). Additionally, it has been proposed that robust expression of SOCS3, induced by IAV infection, contributes to excessive IL-6 production, likely through the hyperactivation of IAV-induced NF-κB signaling (Liu et al., 2019). NF-κB, a key transcription factor, regulates IL-6 production during IAV infection, as shown in several studies (Pahl and Baeuerle, 1995; Liu et al., 2019; Zuo et al., 2021). Unlike IRF3, which is mainly activated by IAV nucleic acids via the MAVS-dependent RIG-I/MDA5 pathway and plays a central role in type I and III interferon production (Liu et al., 2015). NF-κB activation may be predominantly triggered by the sensing of IAV proteins and DAMPs (Pahl and Baeuerle, 1995; Flory et al., 2000; Imai et al., 2008; Tuvim et al., 2012; Holm et al., 2016; Fernández-Arjona et al., 2019; Sun and Metzger, 2019; Kim et al., 2022, Kim et al., 2023), also involving the cGAS-independent STING pathway (Holm et al., 2016), potentially leading to expression of inflammatory cytokines, including IL-6 expression (Kim et al., 2022, Kim et al, 2023). Notably, most IAV proteins, such as PB1-F2, NP, and HA, inhibit RIG-I/TRIM25/MAVS-mediated signaling, thereby impairing type I and III interferon production and subsequent IFN-mediated antiviral responses (Kochs et al., 2007; Iwai et al., 2010; Zeng et al., 2021; Zhang et al., 2023; Ouyang et al., 2024). For example, IAV PB1-F2 binds and inhibits RIG-I/TRIM25/MAVS signaling, suppressing type I and III interferon transcription (Zeng et al., 2021), while NP and HA similarly impair MAVS-mediated antiviral immunity (Zhang et al., 2023; Ouyang et al., 2024). However, while these IAV proteins suppress interferon production, they can also simultaneously activate NF-κB, further contributing to IL-6 expression (Pahl and Baeuerle, 1995; Flory et al., 2000). Moreover, PB1-F2 has been shown to form fibrillar aggregates that promote the release of pyrogenic IL-1β through NLRP3-dependent inflammasome activation, which can amplify IL-6 production by activating NF-κB in both autocrine and paracrine manner (Chanturiya et al., 2004; Zamarin et al., 2006; Pinar et al., 2017; Zeng et al., 2021).

An imbalanced cytokine response, marked by excessive IL-6, correlates with severe disease outcomes in influenza (Gentile et al., 1998; Chiaretti et al., 2013; Zuo et al., 2021; Zhang et al., 2022). A similar dysregulated immune response, marked by low levels of type I and III IFNs alongside elevated chemokines and high IL-6 expression, contributes to the pathogenesis of SARS-CoV-2 infection and the development of COVID-19 (Blanco-Melo et al., 2020). This pattern mirrors observations in severe seasonal influenza, where ferrets exhibited reduced interferon induction and heightened IL-6 levels (Svitek et al., 2008). Interestingly, disruption of STAT3 phosphorylation, a key downstream event in IL-6 signaling, has been shown to result in excessive production of IAV-induced type I and III IFNs (Liu et al., 2023). Additionally, IAV can suppress type I IFN signaling through NF-κB-dependent induction of SOCS3 expression (Pauli et al., 2008; Ye et al., 2015). Despite these findings, the precise molecular mechanisms by which virus-induced IL-6 modulates interferon responses remain unclear. On the other hand, it has been reported that there is competitive inhibition between IRF3 and NF-κB activation (Popli et al., 2022; Chakravarty et al., 2024), underscoring the need for further investigation into the molecular interplay between IL-6 signaling and antiviral IFN pathways during IAV infection.

IL-6 knockout animal models, as described above, indicate that IL-6 depletion impairs both innate and adaptive immune responses, leading to increased morbidity and mortality during influenza infection. However, defining the non-redundant function of IL-6 in influenza virus pathogenesis using these models remains challenging. Some studies have shown that IL-6 knockout mice exhibit similar morbidity and mortality to wild-type mice when infected with highly pathogenic H5N1 influenza virus (Salomon et al., 2007; Szretter et al., 2007).These discrepancies may arise from differences in influenza strain, experimental conditions, or other variables, suggesting a need for further investigation with more rigorous and controlled experimental designs. Interestingly, a recent study has proposed that IL-6 primarily contributes to defense against influenza by promoting protective antibody responses, rather than by mediating innate inflammation (Piattini et al., 2025).

Substantial evidence underscores the pivotal role of IL-6 in viral infections, where it mediates diverse immunological processes. These include the activation of the acute-phase response, modulation of inflammatory cytokine production, induction of chemokine secretion, recruitment of immune cells, and activation of NK cells and cytotoxic T lymphocytes. However, dysregulated IL-6 production can drive a hyperinflammatory state (cytokine storm), which leads to severe pathological consequences for the host. This supports the hypothesis that a finely tuned innate immune response is crucial for effective viral clearance while minimizing immunopathology. In contrast, an alternative perspective posits that disease severity is primarily dictated by viral load rather than immune dysregulation (Wang et al., 2010). For instance, a study utilizing quantitative real-time PCR to assess the cytokine-to-viral RNA ratio found no significant differences between susceptible and resistant mouse strains. These findings suggest that host genetic determinants influencing viral replication and viral load play a dominant role in susceptibility to influenza infection (Wang et al., 2010).

Indeed, elucidating the complex molecular mechanisms underlying host-virus interactions is important for distinguishing between protective and pathological immune responses. To this end, emerging evidence has identified several new host factors as key regulators of innate immunity during influenza virus infection. In particular, non-coding RNAs—including long non-coding RNAs (lncRNA), microRNAs, and circular RNAs—as well as micropeptides have been increasingly recognized as crucial modulators of IAV-induced innate immune responses (Ouyang et al., 2014; Ma et al., 2016; Maarouf et al., 2019; Xiao et al., 2021; Rai et al., 2022; Chen et al., 2024; Chi et al., 2024; Sun et al., 2025). For examples, RDUR, a lncRNA, promotes innate antiviral responses and provides feedback control of NF-κB activation to suppress excessive inflammatory response to IAV infection (Chen et al., 2021b). HOTAIR regulates the activation of NF-κB and its target gene IL-6 expression by facilitating the degradation of IκBα (Obaid et al., 2018). LncRNA MALAT1 regulates inflammatory cytokine production in lipopolysaccharide-stimulated human gingival fibroblasts through sponging miR-20a and activating TLR4 pathways (Li et al., 2020). Targeting of such host factors involved in IAV-induced IL-6 regulation may hold promising therapeutic strategy in the treatment of influenza (Zhang and Chu, 2019). Investigating the roles of these new regulatory elements in innate immune signaling presents exciting avenues for research and may provide critical insights into the molecular mechanisms governing IL-6-mediated cytokine storms.

Given the dual role of IL-6, therapeutic strategies targeting this cytokine should aim to enhance its beneficial antiviral effects while preventing excessive IL-6 production to mitigate tissue damage and multiple organ failure. Especially, the cytokine storm is not solely driven by IL-6 but results from the combined effects of various cytokines and chemokines. Consequently, no single pharmacological intervention has proven entirely effective in preventing tissue damage and inflammation associated with severe influenza. Therefore, it is crucial to explore other cellular pathways, such as apoptosis, pyroptosis, necroptosis, and ferroptosis, which influence innate immune signaling and contribute to cytokine storm pathogenesis (Tripathi et al., 2013; Arora et al., 2016; Shrivastava et al., 2016; Fang and Peng, 2022; Resnik et al., 2023; Ouyang et al., 2024). This is because IAV induces apoptotic, necrotic, and necroptotic death of lung epithelial and pulmonary immune cells (Kim et al., 2023; Boyd et al., 2025). Supportively, a recent study indicated that that blocking cell death (necroptosis blockage) can reduce lung injury in severe influenza by limiting inflammation and tissue damage (Gautam et al., 2024). Z-DNA binding protein 1 (ZBP1) is another innate immune sensor (recently gained a wide research attention) that recognizes Z-RNA generated during IAV-infection leading to RHIM-mediated recruitment and activation of receptor interacting kinase 3 (RIPK3) followed by apoptosis and necroptosis (Oltean et al., 2024). It would be interesting to study the role of IL-6 in ZBP1-initiated necroinflammatory cell death.

## 6 Summary

IL-6 is a key cytokine mediating the innate immune response to IAV, linking innate and adaptive immunity. While optimal IL-6 production appears to exert antiviral defense, excessive IL-6 production can contribute to hyperinflammatory responses and severe disease outcomes, such as ARDS. The molecular mechanisms behind IL-6 excessive production and its contributing to cytokine storm are complex, but evidence suggests that the hyperactivation of inflammatory pathways by DAMPs, PAMPs and IL-6 amplifier contribute to this dysregulation. The magnitude of IL-6 induction depends on a multifactorial interplay of viral factors (e.g., strain specificity, protein polymorphisms, replication kinetics) and host immune responses. Although IL-6 knockout models indicate its importance in host immunity, discrepancies in findings highlight the need for more controlled studies. Emerging research into new host factors like non-coding RNAs and micropeptides that regulate IL-6 is essential to elaborate the precise role of IL-6 in antiviral innate immunity. Given the contribution of IL-6 in cytokine storms, therapeutic strategies require balancing its antiviral effects while preventing excessive production. Future studies are warranted to explore other cellular pathways and processes, such as mitophagy, autophagy, apoptosis, and necroptosis, to better understand how innate immune signaling pathways governing IL-6 induction interact with these processes and mitigate the pathological effects of immune dysregulation and tissue damage during influenza infection.
