# Correction: Innate immune role of IL-6 in influenza a virus pathogenesis

**DOI:** 10.3389/fcimb.2025.1691338

**Published:** 2025-08-28

**Authors:** Xinxin Li, Chen Huang, Kul Raj Rai, Quanming Xu

**Affiliations:** ^1^ Meizhouwan Vocational Technology College, Putian, China; ^2^ Hanjiang District Agriculture and Rural Affairs Bureau, Putian, China; ^3^ Key Laboratory of Fujian-Taiwan Animal Pathogen Biology, College of Animal Sciences, Fujian Agriculture and Forestry University, Fuzhou, China; ^4^ Scientific Research and Experiment Center, Fujian Police College, Fuzhou, China

**Keywords:** IL-6, IAV, innate immunity, inflammation, cytokine storm

In the published article, there was an error in the author affiliations. The affiliations for Chen Huang and Quanming Xu were incorrectly assigned.

Quanming Xu was listed under Affiliation 4: “Hanjiang District Agriculture and Rural Affairs Bureau, Putian, China.” Chen Huang was listed under Affiliation 2: “Scientific Research and Experiment Center, Fujian Police College, Fuzhou, China.”

The correct affiliations should be:

Quanming Xu should be assigned affiliation 2: “Scientific Research and Experiment Center, Fujian Police College, Fuzhou, China”

Chen Huang should be assigned affiliation 4: “Hanjiang District Agriculture and Rural Affairs Bureau, Putian, China”.

The original version of this article has been updated.

