# Supplementary material for: Innate immune role of IL-6 in influenza a virus pathogenesis
Source: Front Cell Infect Microbiol. 2025 Jul 7;15:1605446. doi: 10.3389/fcimb.2025.1605446 (PMC12277322; doi:10.3389/fcimb.2025.1605446)
Supplement: Supplementary file 1 [file Table1.pdf]

Supplementary Material

1 Supplementary Table 1

Host protective function of IL-6				
SN	**STRAIN**	**MODEL**	Mechanisms	
1.	R/8/34 (H1N1)	Mice	Induces long-term protective immunity against a lethal challenge of influenza virus.	(Leethese et al., 1999)
2.	H1N1	Mice	Promotes neutrophil-mediated viral clearance	(Dienz et al., 2012)
3.	PR8	Mice	Enhances anti-viral T-cell response	(Lauder et al., 2013)
4.	A/WSN/33 (H1N1)	Mice	Protects lung epithelial cells from influenza virus-induced apoptosis.	(Yang et al., 2017)
5.	Recombinant IAV strain E61-13-H17 (H17, H3N2) and PR8 (H1N1)	Mice	Limits the activity of virus-specific Tregs, facilitating virus-specific memory CD4+ T-cell activity and pathogen clearance.	(Longhi et al., 2008)
6.	Influenza-*Streptococcus pneumoniae* co-infection	Mice	Protects BALF cells from apoptosis, boosts macrophage function, and prevents secondary bacterial infections.	(Gou et al., 2020)

7.	PR8	Mice	IL-6 directly enhances the expansion and function of memory CD4 T cells during secondary influenza infection.	(Strutt et al., 2016)
		**Detrimental role of IL-6**		
1.	H5N1	Human	Abrupt hypercytokinemia during the initial viral replication in the respiratory tract triggers reactive hemophagocytic syndrome and leads to multiorgan failure in fatal cases.	(Wang et al., 2010)
2.	Avian influenza A (H5N1)	Human	Virus-induced cytokine dysregulation contributes to disease severity and fatal outcome	(Wang et al., 2010)
3.	IAV (A/WSN/33 [H1N1])	Mice	Promotes muscle degradation *via* JAK/STAT, FOXO3a, and atrogin-1 upregulation.	(Radigan et al., 2019)
4.	Pandemic H1N1 influenza A (H1N1pdm)	Humans and mice	IL-6 deficiency does not significantly impact H1N1pdm infection outcome in Mice. IL-6 may serve as an important biomarker for the identification of patients at risk of severe complications following H1N1pdm infection, but may not be a suitable therapeutic target.	(Paquette et al., 2012)
5.	Pandemic H1N1	Human	Intensive cytokine induction in pandemic H1N1 Influenza virus infection accompanied by robust production of IL-10 and IL-6	(Yu et al., 2011)
6.	WSN/33(H1N1)	Mice	Vascular dysfunction in severe influenza in the influenza virus-cytokine-protease cycle axis	(Wang et al., 2010)
7.	H7N9	Human	Study suggests that proinflammatory cytokine (including IL-6) responses, characterized by a combined Th1/Th17 cytokine induction, are partially responsible for the disease progression of patients	(Chi et al., 2013)

8.	PR8	Mice	Early cytokine dysregulation (including IL-6) and viral replication play a role in pulmonary damage and high mortality in lethally infected mice.	(Vogel et al., 2014)
9.	IAV super-infection with *Staphylococcus aureus*	Mice and *in vitro*	Co-infection boosts MAPKs p38 and ERK1/2 activity, leading to excessive IL-6 production.	(Klemm et al., 2017)
10.	IAV	Mice	IAV M1 exacerbated lung pathology and mortality in virus-infected mice in a TLR4-dependent manner, driven by robust expression of proinflammatory cytokines, including IL-6.	(Imai et al., 2008; Kim et al., 2023)
